# Effect of long-term dietary sphingomyelin supplementation on atherosclerosis in mice

**DOI:** 10.1371/journal.pone.0189523

**Published:** 2017-12-14

**Authors:** Rosanna W. S. Chung, Zeneng Wang, Christina A. Bursill, Ben J. Wu, Philip J. Barter, Kerry-Anne Rye

**Affiliations:** 1 Lipid Research Group, Heart Research Institute, Sydney, NSW, Australia; 2 Department of Cellular and Molecular Medicine, Cleveland Clinic, Cleveland, United States of America; 3 South Australian Health and Medical Research Institute, Adelaide, SA, Australia; 4 School of Medical Sciences, Faculty of Medicine, University of New South Wales, Sydney, Australia; Brigham and Women's Hospital, Harvard Medical School, UNITED STATES

## Abstract

Sphingomyelin (SM) levels in the circulation correlate positively with atherosclerosis burden. SM is a ubiquitous component of human diets, but it is unclear if dietary SM increases circulating SM levels. Dietary choline increases atherosclerosis by raising circulating trimethylamine N-oxide (TMAO) levels in mice and humans. As SM has a choline head group, we ask in this study if dietary SM accelerates atherosclerotic lesion development by increasing circulating SM and TMAO levels. Three studies were performed: (Study 1) C57BL/6 mice were maintained on a high fat diet with or without SM supplementation for 4 weeks prior to quantification of serum TMAO and SM levels; (Study 2) atherosclerosis was studied in apoE^-/-^ mice after 16 weeks of a high fat diet without or with SM supplementation and (Study 3) apoE^-/-^ mice were maintained on a chow diet for 19 weeks without or with SM supplementation and antibiotic treatment prior to quantification of atherosclerotic lesions and serum TMAO and SM levels. SM consumption did not increase circulating SM levels or atherosclerosis in high fat-fed apoE^-/-^ mice. Serum TMAO levels in C57BL/6 mice were low and had no effect atherosclerosis lesion development. Dietary SM supplementation significantly reduced atherosclerotic lesion area in the aortic arch of chow-fed apoE^-/-^ mice. This study establishes that dietary SM does not affect circulating SM levels or increase atherosclerosis in high fat-fed apoE^-/-^ mice, but it is anti-atherogenic in chow-fed apoE^-/-^ mice.

## Introduction

Sphingomyelin (SM), a ubiquitous component of the human diet, is abundant in dairy products, eggs and meats [1, 2]. Dietary supplementation with egg SM reduces plasma cholesterol and triglyceride levels in hyperlipidemic APOE*3 Leiden mice [3]. It also decreases hepatic steatosis in Zucker fatty rats [4]. We have recently reported that a high-fat diet supplemented with SM dose-dependently reduces hepatic steatosis in C57BL/6 mice [5]. As hyperlipidemia and hepatic steatosis are major risk factors for atherosclerosis, these observations suggest that dietary SM may be anti-atherogenic.

However, there is evidence that elevated circulating SM levels are atherogenic in humans [6, 7] and mice [8–10]. Dietary SM is metabolized in the intestine and enters the circulation as a component of chylomicrons. However, the impact of dietary SM on circulating SM levels has not been investigated systematically [11]. Therefore, we asked if dietary SM directly increases circulating SM levels and atherosclerotic lesion development.

Previous reports have established that dietary phosphatidylcholine is metabolized by gut flora into choline and trimethylamine *N*-oxide (TMAO), both of which increase atherosclerosis in apolipoprotein E knockout mice (apoE^-/-^ mice) [12]. Elevated plasma TMAO levels are also associated with increased risk of major CVD events in humans [13]. As SM also contains a choline head group, the possibility that it accelerates atherosclerosis by increasing circulating levels of TMAO, is addressed in the present study.

To determine if dietary SM is converted into TMAO, plasma TMAO levels were quantified in C57BL6 mice fed a high fat diet supplemented with escalating amounts of SM (Study 1). The effect of a high fat diet with SM supplementation on atherosclerotic lesion development was assessed in apoE^-/-^ mice in (Study 2). As intestinal absorption of SM can be inhibited by dietary cholesterol, the effect of dietary SM supplementation on atherosclerotic lesion development and TMAO levels was also assessed in chow-fed apoE^-/-^ mice (Study 3).

## Materials and methods

### Animal models

All animal experiments were approved by the Sydney South West Area Health Service Animal Welfare Committee (Protocol Number 2011/020). The animals were housed in standard cages (3–5 mice per cage) at 21 ^o^C with a 12 h light/dark cycle and *ad libitum* access to food and water. Food intake and body weight were monitored twice weekly. For euthanasia, mice were subjected to exsanguination by cardiac puncture under methoxyfluorane anesthesia after a 12-hour overnight fast. Body weight was recorded before and after fasting.

#### Study 1

The effect of dietary SM supplementation on circulating choline and TMAO levels was determined in four groups of 4-week-old male C57BL/6 mice (Monash University, Melbourne, Victoria) that were placed on a high fat, semi-purified diet (SP00-219, Specialty Feeds, Glen Forrest, Western Australia) without (n = 8) or with 0.3% (n = 10), 0.6% (n = 10) or 1.2% (wt/wt) (n = 10) pure chicken egg-derived SM (Lipoid, Ludwigshafen, Germany) for 4 weeks (Table 1). The SM-enriched diets were prepared by substituting the butterfat component with an equivalent weight of egg SM. We have previously shown that these SM concentrations decrease high fat diet-induced hepatic steatosis in C57BL/6 mice [5].

**Table 1:** Design of animal studies.

	Study 1	Study 2	Study 3
**Aim**	Effect of dietary SM on TMAO, betaine and choline levels	Effect of dietary SM on atherosclerosis and circulating SM levels	Effect of dietary SM on atherosclerosis and circulating SM, betaine and TMAO levels
**Species**	C57BL/6 mice	ApoE^-/-^ mice	ApoE^-/-^ mice
**Groups**	1. HFD 2. HFD+0.3% SM 3. HFD+0.6% SM 4. HFD+1.2% SM	1. HFD 2. HFD+1.2% SM	1. N 2. N+1.2% SM 3. N+antibiotics 4. N+1.2% SM+antibiotics
**Duration**	4 weeks	16 weeks	19 weeks
**Analysis**	TMAO, choline and betaine levels	Aortic lesions Serum lipid levels	Aortic lesions Lesion composition Serum lipid, TMAO, choline and betaine levels

HFD: high fat diet; N: normal chow diet; SM: sphingomyelin

The high fat diet contained 21% (wt/wt) butterfat and 0.15% (wt/wt) cholesterol. Its composition was: casein, 195 g/kg; DL-methionine, 3 g/kg; sucrose 341 g/kg; wheat starch, 154 g/kg; cellulose, 50 g/kg; clarified butter, 210 g/kg; calcium carbonate, 17.1 g/kg; sodium chloride, 2.6 g/kg; potassium citrate, 2.6 g/kg; potassium dihydrogen phosphate, 6.9 g/kg; potassium sulphate, 1.6 g/kg; AIN93G trace minerals, 1.4 g/kg; choline chloride (75%), 2.5 g/kg; vitamins, 10 g/kg; cholesterol, 1.5 g/kg.

#### Study 2

The effect of dietary SM on atherosclerosis development was investigated for the first time in 5-week old female apoE^-/-^ mice (C57BL6 background, Biological Facility, Heart Research Institute, Sydney, Australia). Female apoE^-/-^ mice were used for this study, and Study 3, because they are more susceptible to atherosclerosis and have higher circulating TMAO levels than male mice [12]. The animals were fed either a high fat diet (n = 12) or a high fat diet supplemented with 1.2% (wt/wt) pure SM (n = 9) for 16 weeks (Table 1). The diets were prepared by substituting fat with an equivalent weight of pure egg SM (BOC Sciences, USA).

The high fat diet contained 0.18% (wt/wt) choline. This was unlikely to have influenced the outcomes of Study 1 or Study 2 because all mice had the same dietary choline intake.

#### Study 3

To further investigate the effect of dietary SM on atherosclerosis in the absence of the potentially confounding effects of a high fat diet, female apoE^-/-^ mice were randomly allocated into four groups and maintained for 19 weeks on a: (i) normal chow diet (n = 10), (ii) normal chow diet supplemented with 1.2% (wt/wt) egg SM (n = 9), (iii) normal chow diet plus antibiotics (n = 10), or (iv) normal chow diet supplemented with 1.2% (wt/wt) egg SM and antibiotics (n = 12) (Table 1).The SM-supplemented diet was prepared by adding 1.2% (wt/wt) egg SM (BOC Sciences, USA) to normal chow.

The chow diet was composed of wheat, lupins, barley, soya meal, mixed vegetable oils, canola oil, sodium chloride, calcium carbonate, di-calcium phosphate, magnesium oxide and a vitamin and trace mineral premix. It contained 4.8% total fat, 4.8% crude fibre, 20% protein, 7.6% acid detergent fibre, 16.4% neutral detergent fibre and 59.4% digestible energy.

The antibiotic solution was prepared by dissolving 1 g/L neomycin sulfate (Biomatik Corporation, Canada), 1 g/L ampicillin (MP Biomedicals, Australia), 1 g/L metronidazole (MP Biomedicals, Australia), and 0.5 g/L vancomycin HCl (Biomatik Corporation, Canada) in autoclaved water. The solution was filtered (0.22 μm) and stored for a maximum of 2 weeks at 4°C before use.

### Tissue collection

All animals were exsanguinated by cardiac puncture under methoxyflurane anaesthesia. Serum was collected and stored at -80°C until analysis. After exsanguination, the animals were perfused with 10% (v/v) neutral buffered formalin (Sigma-Aldrich, USA). The heart and the entire aorta were fixed in 4% (v/v) paraformaldehyde and 10% (v/v) neutral buffered formalin, respectively, and stored at 4°C until analysis.

### Quantification of atherosclerotic lesion area

Connective and fat tissue was removed from the aortas prior to longitudinal dissection. The aortas were stained for 5 min with 0.5% (wt/v) Oil Red O in 60% (v/v) aqueous triethyl phosphate, rinsed in 60% (v/v) triethyl phosphate, then rinsed in distilled water. Aortic lesion area was quantified using ImageJ.

Lesion size and protein expression in the aortic sinus were quantified in paraffin-embedded sections. Aortic sinus cross-sections were stained with haematoxylin and eosin and lesion area was quantified with ImageJ. Aortic sinus sections were also immunostained for Mac-3 (1:200 dilution, BD Biosciences) and CD36 (1:50 dilution, Abcam) and amplified using the Vectastain ABC kit (Vector Laboratories) and Envision+ System-HRP (Dako) respectively according to manufacturer’s instructions. Both proteins were visualized with the DAB Peroxidase Substrate Kit (Vector Laboratories). Smooth muscle cells were stained with α-actin antibodies conjugated to alkaline phosphatase (1:100 dilution, Sigma Aldrich) and visualized with the Alkaline Phosphatase Substrate Kit (Vector Laboratories).

### Quantification of serum TMAO, choline and betaine levels

Serum choline, TMAO and betaine levels were quantified by LC-MS/MS with stable isotope dilution as described [12]. Characteristic precursor-product ion transitions of TMAO (*m/z* 76 → 58), choline (*m/z* 104 → 60) and betaine (*m/z* 118 → 59) were monitored in positive MRM MS mode. The internal standards TMAO-trimethyl-d_9_ (d9-TMAO) and choline-trimethyl-d_9_ (d_9_-choline) were added to the serum samples before protein precipitation and monitored in MRM mode at *m/z* 85 → 68 and *m/z* 113 → 69, respectively. Calibration curves for TMAO quantification were established using a spiked control sample with various concentrations of TMAO and fixed amounts of internal standards.

### Quantification of serum glucose and serum lipids

Serum total cholesterol, and triglyceride levels were assayed enzymatically using commercially available kits and standards (Roche Diagnostics, Switzerland). Non-esterified fatty acids levels were measured using NEFA C kits (Wako Pure Chemicals, Japan). Serum SM levels were quantified with a sphingomyelin assay kit (Cayman, USA).

### Statistical analysis

Variance between groups was tested by Bartlett’s test for normal variance and significant differences between multiple groups were assessed by one-way ANOVA using GraphPad Prism (version 6.0, GraphPad Software, Inc.). Student’s *t*-test (homoscedastic, two-tailed) was used when comparing two groups. In case of nonparametric data, multiple-group and two-group comparison were performed using Kruskal-Wallis Test and Mann-Whitney Test respectively in SPSS. The results are reported as the mean ± SEM, with a p-value of <0.5 being considered statistically significant.

## Results

We have previously shown that SM supplementation dose-dependently decreases high fat diet-induced hepatic steatosis in male C57BL/6 mice [5]. Since hepatic steatosis is a major risk factor for atherosclerosis in mice, this raises the possibility that dietary SM may be anti-atherogenic. However, as SM contains a choline head group which could be metabolized into pro-atherogenic compounds such as TMAO, choline and betaine, we used the previously published mouse model [5] to determine if dietary SM affects circulating TMAO, choline and betaine levels (Study 1, Table 1).

Initial body weight, weight gain and food intake were comparable in all the mice in Study 1 (Table 2). No adverse effects from the high fat diet, or the high fat diet supplemented with SM, were observed. This is consistent with what we have reported previously [5].

**Table 2:** Body weight, food intake and serum choline and TMAO levels in mice fed a high fat diet without or with SM supplementation (Study 1).

Diet	HF	HFSM 0.3%	HFSM 0.6%	HFSM 1.2%
**I Initial body weight (g)**	17.25 ± 0.63	17.26 ± 0.43	16.78 ± 0.78	16.71 ± 0.55
**Final body weight (g)**	25.95 ± 0.94	25.46 ± 0.51	24.00 ± 0.54	25.07 ± 0.59
**Food intake (g/ day)**	3.44 ± 0.14	3.30 ± 0.14	3.29 ± 0.16	3.38 ± 0.12
**Serum TMAO (μmol/L)**	0.72 ± 0.07	0.67 ± 0.10	0.56 ± 0.06	1.46 ± 0.15***
**Serum Choline (μmol/L)**	18.64 ± 1.25	19.01 ± 1.48	17.75 ± 1.36	19.76 ± 0.92
**Serum Betaine (μmol/L)**	77.96 ± 12.34	71.22 ± 7.37	56.39 ± 4.02	82.09 ± 8.26

Male C57BL/6 mice (n = 8-10/group) were fed a high fat (HF) diet without or with 0.3%, 0.6% or 1.2% (wt/wt) SM supplementation (HFSM) for 4 weeks. Body weight and food intake were recorded twice weekly. Serum levels of TMAO, choline, and betaine were quantified as described in Materials and Methods. Values represent the mean ± SEM. Significant differences between the HF and HFSM groups were determined by one-way ANOVA: ***P<0.001.

Serum TMAO levels were increased in high fat-fed C57BL/6 mice supplemented with 1.2% (wt/wt) SM relative to the mice that received the high fat diet only (0.72 ± 0.07 vs 1.46 ± 0.15 μmol/L, *P*<0.001), but not in the mice supplemented with 0.3 and 0.6% (wt/wt) SM (Table 2). As the SM-derived TMAO levels in the high fat-fed mice supplemented with 1.2% (wt/wt) SM were very low [12], it is unlikely that this increase would impact on atherosclerosis susceptibility. Dietary SM supplementation did not affect serum choline or betaine levels (Table 2).

To determine if dietary SM increases atherosclerosis, atherosclerosis-prone female apoE^-/-^ mice were fed a high fat diet with or without 1.2% (wt/wt) SM supplementation for 16 weeks (Study 2). The decision to use 1.2% (wt/wt) SM supplementation in Studies 2 and 3, was based on the outcome from Study 1, in which this dose was well tolerated. The mice in Study 2 had comparable body weights at baseline (Table 3). After the dietary intervention, the high fat-fed SM-supplemented mice weighed significantly more than the control, high fat-fed mice (21.16 ±0.51 g vs 18.76±0.43 g, Table 3), (*P*<0.01). SM supplementation had no effect on liver weight (1.0 ± 0.021 vs 1.0 ± 0.022 g for high fat-fed apoE^-/-^ mice without and with SM supplementation, respectively).

**Table 3:** The effect of dietary SM supplementation on body weight and serum lipid levels in high fat-fed apoE^-/-^ mice (Study 2).

Diet	HF	HFSM 1.2%
**Initial body weight (g)**	15.88±0.33	15.55±0.44
**Final body weight (g)**	18.76±0.43	21.16±0.51**
**Weight gain (g)**	2.89±0.54	5.61±0.29***
**Serum cholesterol (mmol/L)**	40.95±3.87	33.31±2.69
**Serum triglycerides (mmol/L)**	1.34±0.14	1.68±0.17
**Serum NEFA (mEq/L)**	1.11±0.14	1.32±0.20
**Serum SM (mg/dL)**	149.85±13.16	151.53±4.79

ApoE^-/-^ mice were maintained on a high fat diet with or without SM for 16 weeks. Serum lipid levels were quantified as described in Materials and Methods. The values represent the mean ± SEM. Significant differences between high fat-fed (HF) animals and the high fat-fed, SM-supplemented animals (HFSM) were determined using a Student’s t-test (homoscedastic, two-tailed). NEFA: Non-esterified fatty acid.

**P<0.01

***P<0.001

Dietary SM supplementation did not affect serum cholesterol, triglyceride, non-esterified fatty acid (NEFA) or SM levels (Table 3). This indicates that dietary SM supplementation does not cause dyslipidemia in high fat-fed apoE^-/-^ mice, which is in agreement with our previous report [5].

**Fig 1:**
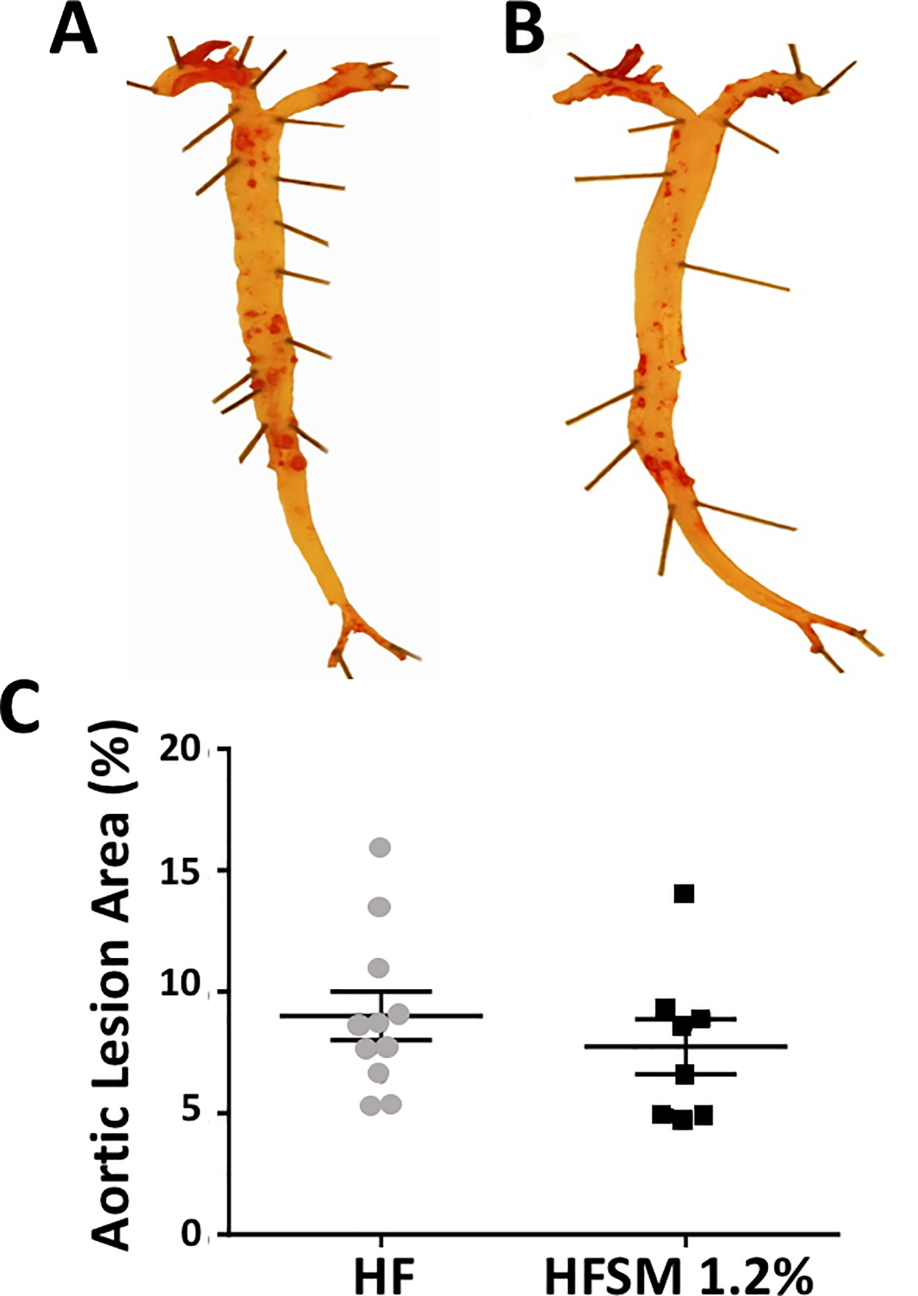
The effect of dietary SM on aortic lesion area in apoE^-/-^ mice fed a high-fat diet without or with SM. ApoE^-/-^ mice were maintained for 16 weeks on a high-fat diet without (Panel A, HF, n = 12) or with 1.2% (wt/wt) SM (Panel B, HFSM 1.2%, n = 9). Representative stained aortas are shown. Panel C: Oil Red O staining. Each data point represents a single animal. The mean ± SEM is shown.

*En face* Oil Red O staining of the aortas in the high fat-fed apoE^-/-^ mice established that lesions constituted 9.0±1.0% of the total aortic area (Fig 1A and 1C), compared to 7.8±1.1% in the high fat-fed mice that received dietary SM supplementation (Fig 1B and 1C). As this difference did not reach statistical significance, it follows that dietary SM does not affect atherosclerotic lesion development in high fat-fed apoE^-/-^ mice.

As the high fat diet used in Study 1 and Study 2 contained a high level of cholesterol, that may have reduced SM bioavailability [14], it was decided to further assess the atherogenicity of dietary SM in mice fed a normal chow diet. ApoE^-/-^ mice were maintained on a normal chow diet with or without SM supplementation for 19 weeks (Study 3, Table 1). Half of the animals were also treated with antibiotics to determine if depletion of gut flora, which decreases TMAO production, attenuated atherosclerotic lesion development [12]. Neither dietary SM supplementation, nor treatment with antibiotics alone, affected body weight in chow fed apoE^-/-^ mice (data not shown).

**Fig 2:**
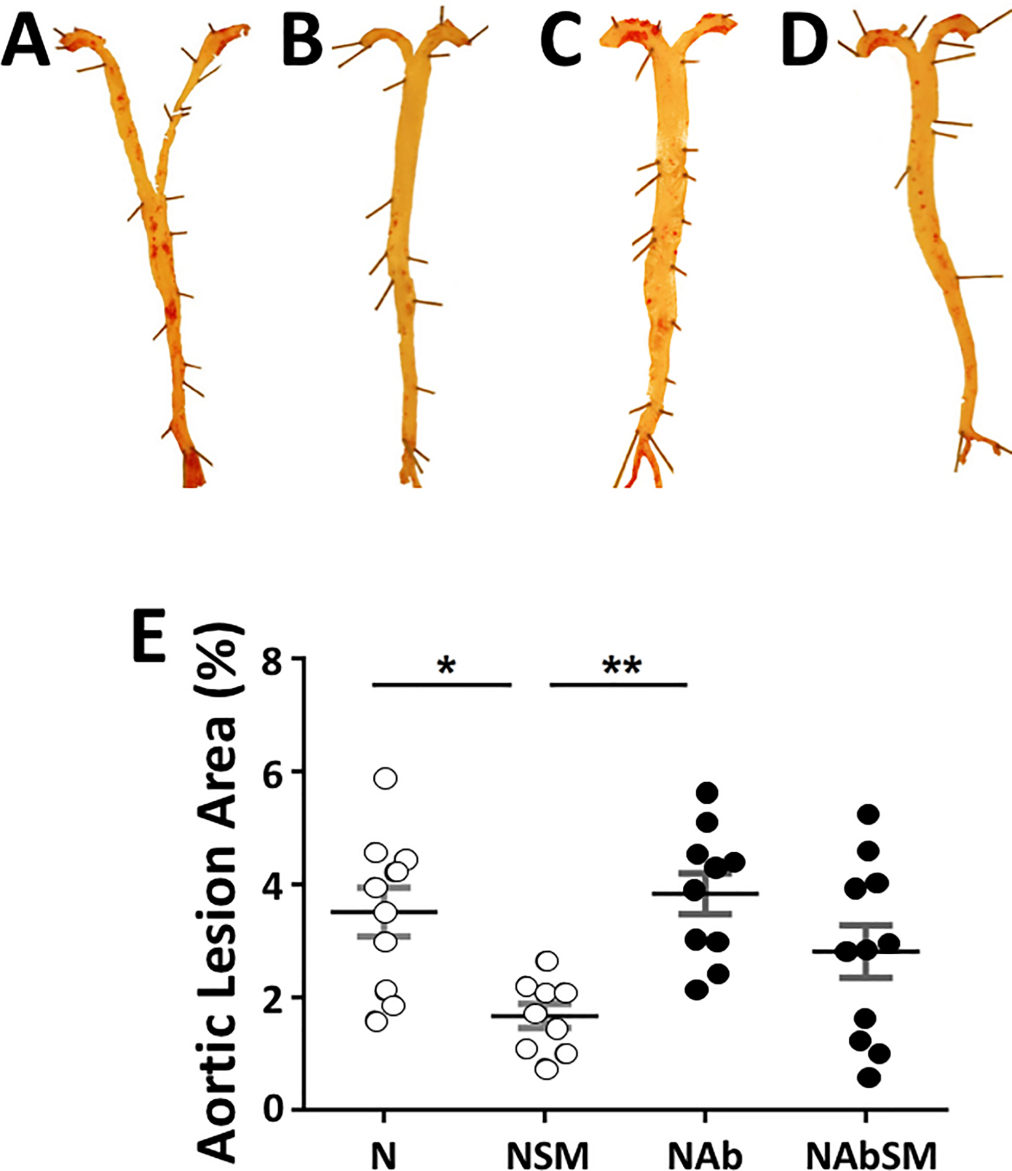
The effect of dietary SM on aortic lesion area in apoE^-/-^ mice fed a normal chow diet with or without SM and antibiotic supplementation. ApoE^-/-^ mice were maintained for 19 weeks on normal chow (Panel A, N, n = 10), chow supplemented with SM (Panel B, NSM, n = 9), chow plus antibiotics (Panel C, NAb, n = 10) or chow with SM and antibiotics (Panel D, NAbSM, n = 12). Oil red O staining was quantified with ImageJ. Representative stained aortas are shown (Panes A-D). Each data point represents a single animal (Panel E). The mean ± SEM is shown. Significant differences between groups were determined by one-way ANOVA. *P<0.05, **P<0.01.

*En face* quantification of atherosclerotic lesions with Oil Red O staining established that SM supplementation decreased lesion area. Oil Red O staining comprised 3.51±0.43% of the total aortic surface in the chow-fed animals (Fig 2A and 2E), compared to 1.67±0.21% in the chow-fed animals that received dietary supplementation with SM (*P*<0.05) (Fig 2B and 2E). The animals that received SM (Fig 2B) also had fewer lesions than the animals treated only with antibiotics (Fig 2C and 2E) (*P*<0.01). The reduction in lesion area was mostly confined to the aortic arch. Lesion area was comparable in the chow-fed (N), the antibiotic-treated (Ab) animals and in the animals treated with antibiotics and SM (NAbSM).

**Fig 3:**
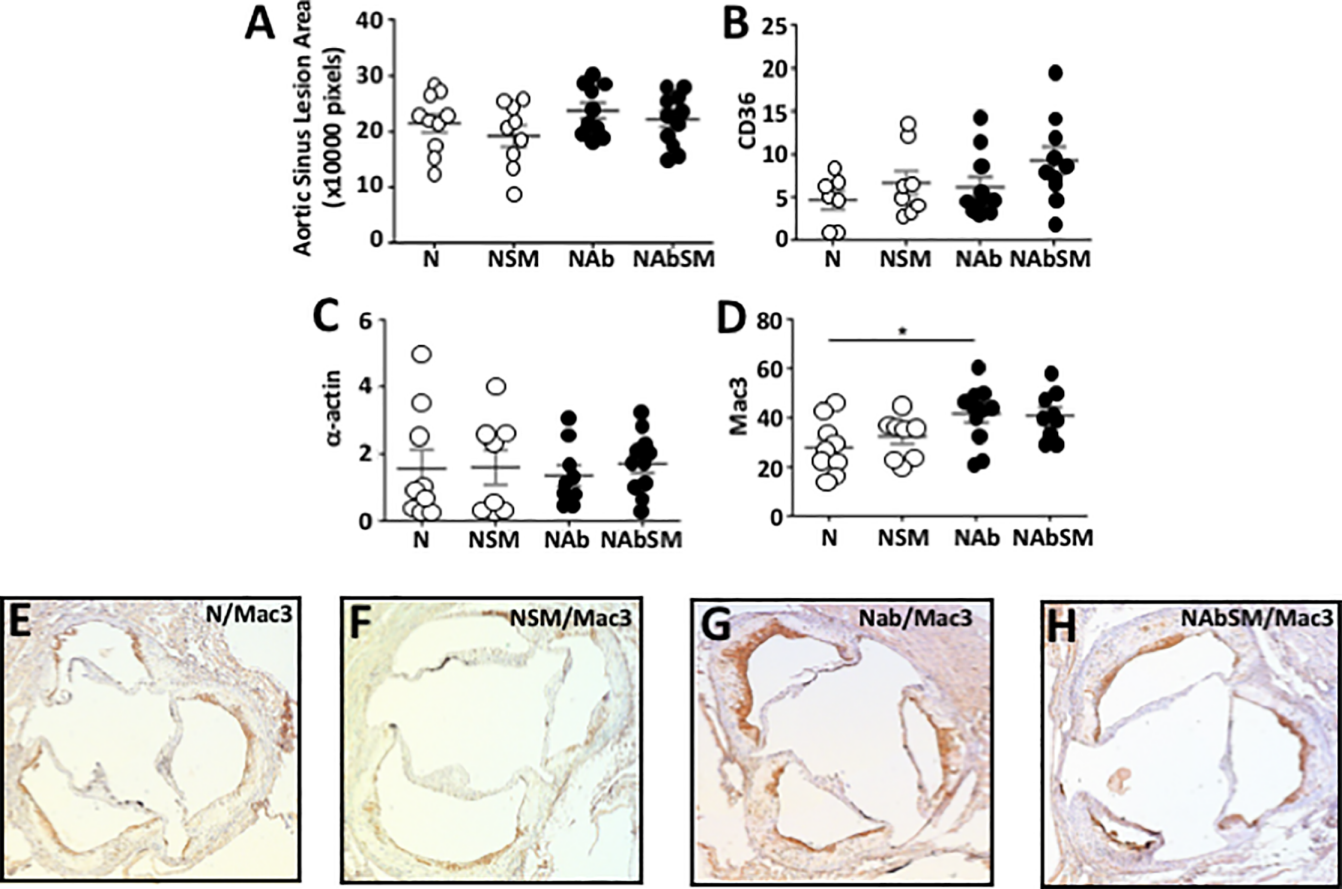
Dietary SM does not affect atherosclerosis in apoE^-/-^ mice. ApoE^-/-^ mice were maintained for 19 weeks on chow (N, n = 10), chow supplemented with SM (NSM, n = 9), chow plus antibiotics (NAb, n = 10) or chow supplemented with SM and antibiotics (NAbSM, n = 12). The aortic sinus was stained with haematoxylin and eosin, and quantified with ImageJ (Panel A). CD36 (Panel B), α-actin (Panel C) and α-Mac3 (Panel D) levels in the aortic sinus were quantified immunohistochemically. Representative Mac-3 staining in the aortic sinus from mice fed chow (Panel E), chow supplemented with SM (Panel F), chow plus antibiotics (Panel G) and chow supplemented with SM and antibiotics (Panel H) are shown. Each point in Panels A-D represents data from a single animal. Error bars represent means ± SEM. Significant differences between groups were determined by one-way ANOVA: ** *P*<0.05 NAb versus N.

Atherosclerotic lesion area in the aortic sinus was quantified, but no difference was found between groups (Fig 3A). Lesion composition in the aortic sinus was assessed immunohistochemically. Dietary SM supplementation did not affect aortic sinus CD36 (Fig 3B) or α-actin expression (Fig 3C). Mac3 expression was, by contrast, significantly increased in the antibiotic-treated animals (Fig 3D–3F vs Fig 3D and 3G–3H) relative to the animals that did not receive antibiotics (*P*<0.05). SM supplementation did not affect lesion macrophage content in the aortic sinus, irrespective of whether or not the animals were treated with antibiotics.

**Fig 4:**
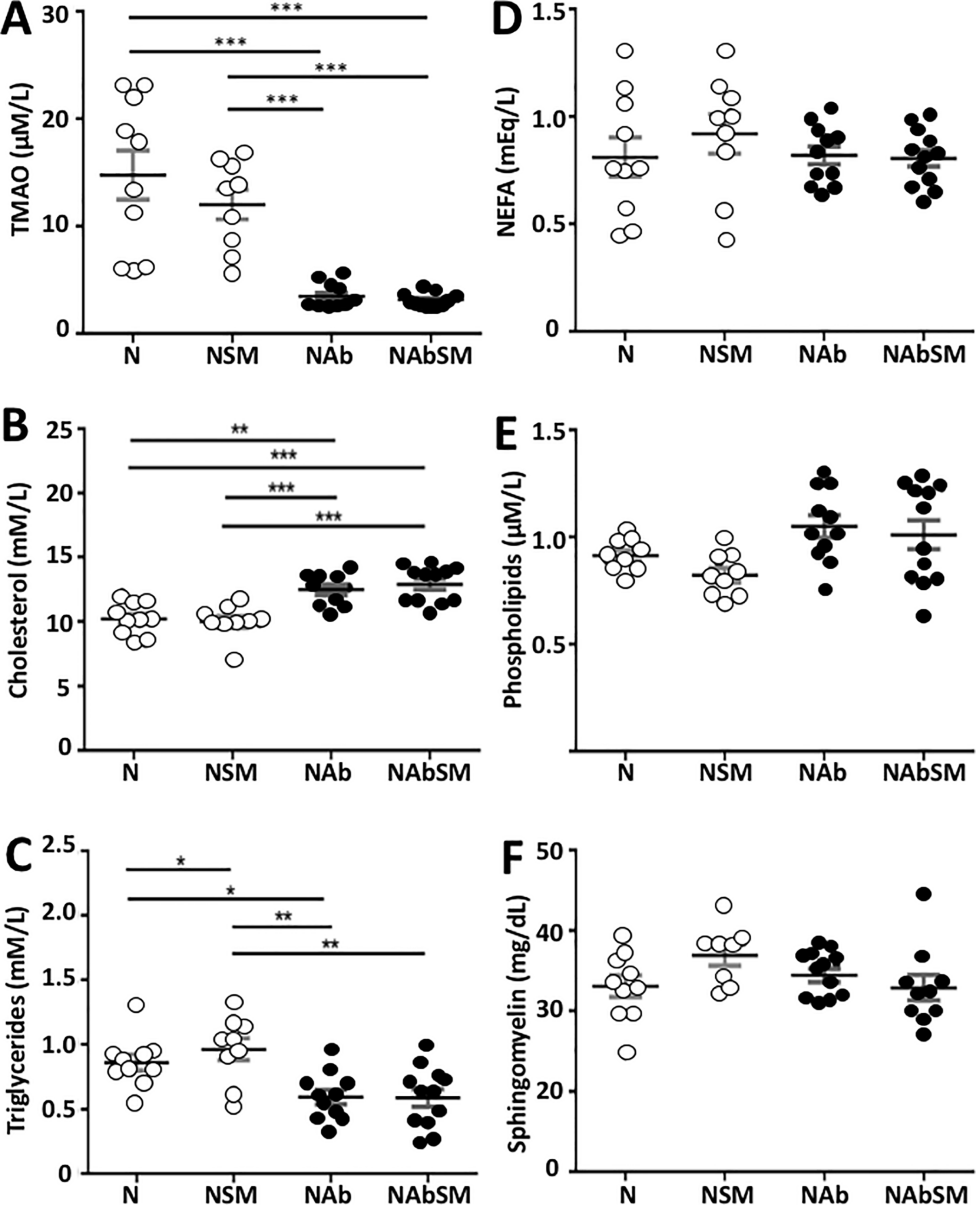
The effect of dietary SM on serum lipids and TMAO levels in mice maintained on chow with or without antibiotics. ApoE^-/-^ mice were maintained on chow (N, n = 10), chow supplemented with SM (NSM, n = 9), chow plus antibiotics (NAb, n = 10) or chow supplemented with SM and antibiotics (NAbSM, n = 12) for 19 weeks. Levels of serum TMAO (Panel A), cholesterol (Panel B), triglycerides (Panel C), NEFA (Panel D), phospholipids (Panel E), and SM (Panel F) are shown. Each point represents data from a single animal. Error bars are presented as the means ± SEM. Significant differences between groups were determined by one-way ANOVA:*P<0.05, **P<0.01, ***P<0.001, or with the Mann-Whitney Test: NEFA: Non-esterified fatty acids. Significant differences between groups were determined.

The effect of dietary SM and antibiotic treatment on serum TMAO and lipid levels in was also quantified. Nineteen weeks of dietary SM supplementation did not increase serum TMAO levels (Fig 4A). While this result differs from what was found for the C57BL/6 mice in Study 1, it is important to note that C57BL/6 mice have much lower serum TMAO than apoE^-/-^ mice (0.7 μM/L vs 15 μM/L) and the SM-mediated increase in TMAO levels of 0.74 μM/L that was observed in Study 1 will not impact significantly on the overall TMAO level in SM-supplemented apoE^-/-^ mice.

This indicates that dietary SM is not metabolized by gut flora in chow-fed apoE^-/-^ mice. Antibiotic treatment, by contrast, reduced serum TMAO levels approximately 3-fold, irrespective of whether the mice were maintained on normal or SM-supplemented chow (Fig 4A, *P*<0.001). This confirms that antibiotic treatment depletes gut flora and decreases TMAO formation [12].

Serum cholesterol (Fig 4B) and triglyceride levels (Fig 4C) were not affected by dietary SM. However, serum cholesterol levels were increased (Fig 4B) and serum triglyceride levels were reduced (Fig 4C) by antibiotic treatment. Finally, dietary supplementation with SM and antibiotic treatment had no effect on serum NEFA, phospholipid or SM levels in chow fed apoE^-/-^ mice (Fig 4D–4F).

## Discussion

Endogenous SM has been reported by many investigators to be pro-atherogenic [7, 15], and high circulating SM levels in humans are associated with increased cardiovascular risk [16]. To date, there is no evidence that dietary SM levels affects circulating SM levels. In contrast to the documented pro-atherogenic effects of endogenous SM, we have previously shown that dietary supplementation of high fat-fed C57BL/6 mice with 1.2% (wt/wt) SM for 4 weeks dose-dependently decreases diet-induced hepatomegaly and hepatic steatosis, both of which are associated with increased cardiovascular risk, [5]. In the present study, we extend these findings by establishing that long term dietary supplementation with SM does not increase atherosclerosis in high fat-fed, and decreases atherosclerotic lesion development in the aortic arch of chow-fed apoE^-/-^ mice.

The inability of dietary SM to increase circulating SM levels may be a reflection of poor intestinal SM absorption. Around 25% of dietary SM is excreted [11], with the remainder being metabolized and absorbed as free fatty acids and sphingosine [17–19]. The absorbed sphingosine is subsequently incorporated into the sphingolipid pool, where it is used for *de novo* sphingolipid synthesis. Multiple studies have reported that the level of SM synthase, the key enzyme in the *de novo* sphingolipid synthesis pathway, is the major determinant of SM levels in the liver, plasma and macrophages [15, 20, 21]. Deficiency of SM synthase decreases plasma SM levels in mice by more than 50% [15]. There is, by contrast, no evidence that increasing dietary SM intake stimulates *de novo* sphingolipid biosynthesis and increases plasma SM levels. The results from this study support the notion that dietary SM intake is not a determinant of plasma SM levels in mice.

We also tested the possibility that the choline headgroup of dietary SM is metabolized by gut flora to TMAO, which is pro-atherogenic. Circulating TMAO levels are positively correlated with increased cardiovascular risk in humans and accelerated atherosclerosis in mice [12]. Dietary choline, phosphatidylcholine and L-carnitine, all of which are TMAO precursors, have been implicated in this effect, and dietary supplementation with these compounds significantly increases serum TMAO levels and atherosclerosis in mice and humans [12, 13]. Although serum TMAO levels in high fat-fed C57BL/6 mice supplemented with 1.2% (wt/wt) SM were significantly higher than in control mice, the TMAO levels in both groups were very low relative to the levels reported by Wang *et*.*al*. [12], and unlikely to impact on atherosclerotic lesion development. This is also consistent with the results in high fat-fed apoE^-/-^ mice in Study 2, where 16 weeks of dietary supplementation with SM did not affect atherosclerotic lesion development, and with the report of Tang *et al*. who did not find a significant correlation between plasma TMAO levels and major adverse cardiovascular events in patients with TMAO levels in the lower quartile, whereas TMAO levels were predictive of major cardiovascular events in patients with high TMAO levels [13].

As dietary SM can form a complex with cholesterol, which reduces the bioavailability of free SM to gut flora for TMAO production, we also asked if dietary SM supplementation would lead to higher serum TMAO levels in apoE^-/-^ mice maintained on a normal chow diet that has a low cholesterol content. Although no effect on serum TMAO levels was observed in this study, atherosclerotic lesion development in the aortic arch was decreased in the chow-fed apoE^-/-^ mice relative to control mice. The underlying mechanism of this observation is not clear, but as circulating lipid and TMAO levels were not affected by the SM supplementation, it is possible that the dietary SM modulated gut flora composition in the chow-fed mice in a way that reduces atherosclerosis. A recent study reported that 4 weeks of dietary supplementation with 0.25% (wt/wt) SM selectively increases the abundance of *Bifidobacterium* and attenuates obesity, metabolic syndrome, and macrophage activation in C57BL/6 mice [22, 23]. The significant changes in serum cholesterol and triglyceride levels that were observed in the antibiotic-treated animals in the present study are consistent with what has been observed by other investigators [24, 25]. Although the underlying mechanism of this effect is unclear, it may be due to changes in gut flora that play a pivotal role in nutrient digestion and lipid homeostasis. Finally, it should be noted that the animals in the present study consumed a much higher level of SM (approximately 1.8 g/kg/day) than the average dietary SM intake in humans (4–6 mg SM/kg/day) [26].

In conclusion, this study establishes that dietary SM does not affect circulating SM levels or increase atherosclerosis in mice that are maintained on a high fat diet, but that it is anti-atherogenic in chow-fed apoE^-/-^ mice, possibly due to SM-mediated alterations in gut flora. This possibility is worthy of further investigation.
